# Institutional Questions

**Published:** 1899-12-09

**Authors:** 


### INSTITUTIONAL QUESTIONS.

### Paraffin Wax Process for Floors.

"Cottage Hospital Secretary" writes: Can you supply me with the name and address of any firm from whom I could obtain materials, appliances, and instructions for preparing floors with the paraffin wax process?

⁂ We should advise you to apply to any builder in your neighbourhood, who would be certainly able to do what is required for the floors of your hospital. It would not be satisfactory to attempt carrying out the treatment it is proposed to apply except by someone understanding the work.

### Hospital Kitchens.

"Architect" asks: It is proposed to erect new central kitchens in a large institution for incurables in Australasia. I should be greatly obliged to you if you could forward me any information or plans relating to modern buildings of this class and their necessary appliances. It is not easy in Australia to obtain such information, but by communicating with the fountain head of such knowledge I trust a satisfactory result will ensue.

⁂ You will find a plan of a model central kitchen and its appurtenances in the portfolio of plans published with Sir Henry Burdett's "Hospitals and Asylums of the World," pages 14 and 15. On page 14, ground floor plan, front administration block, you will find behind the main block on the ground floor the general store, linen store, bedding store, linen mending-room, receiving-room, nurses' dining-room, servants' hall, &c. On the first floor plan you will find the kitchen, scullery, larder, bread and grocery stores, and other necessary offices. It would hardly be possible to find a plan more carefully thought out, or one which in practice has proved so adequate, complete, and excellent for working purposes as this kitchen plan of the Derbyshire Royal Infirmary. The kitchen arrangements are excellent, both from an architectural and administrative point of view. You do not state the number of beds in the institution you refer to. The Derbyshire Infirmary contains 198. There are no features of special interest attaching to the kitchen arrangements of any existing hospitals for incurables. In volume IV. of "Hospitals and Asylums" you will find a full description of planning kitchen offices and all that appertains to them. You would probably find a copy of this book in any large public library.

